# Understanding the role of climatic and environmental variables on the population dynamics of *Anopheles gambiae* s.s. and the implications for vector control strategies in different settings

**DOI:** 10.1186/1475-2875-11-S1-P76

**Published:** 2012-10-15

**Authors:** Paul E Parham, Diane Pople, Céline Christiansen-Jucht, Steve Lindsay, Wes Hinsley, Edwin Michael

**Affiliations:** 1 Grantham Institute for Climate Change, Department of Infectious Disease Epidemiology, Imperial College London, W2 1PG, UK; 2 Department of Infectious Disease Epidemiology, Imperial College London, W2 1PG, UK; 3 Disease Control and Vector Biology Unit, London School of Hygiene and Tropical Medicine, London, WC1E 7HT, UK; 4 Department of Biological Sciences, University of Notre Dame, Notre Dame, IN 46556-0369, USA

## Background

The impact of weather, climate, and environmental conditions on malaria transmission have attracted increasing attention in recent years, although there remain significant uncertainties and debate regarding its precise role. Global mean temperatures are predicted to increase by several degrees over the coming century, although increases in Africa, accounting for over 90% of the global malaria burden, are expected to be greater than the global mean, with anticipated median increases around 3-4°C (depending on the emissions scenario). Global mean precipitation is also predicted to increase in most emissions scenarios over the coming decades, although there is currently less consensus between climate models on expected changes thereafter, particularly at low latitudes where the majority of cases arise and across many regions of Africa. Mathematical models provide powerful tools for understanding the impact of changes in environmental variables on transmission and intervention strategies, but this first requires realistic modelling of *Anopheles* population dynamics and its response to environmental variables.

## Materials and methods

Experimental and field data are used to develop new parameterisations to model the relationships between key aspects of *An. gambiae* s.s. ecology and climatic and environmental conditions. These relationships are integrated into a deterministic model of *Anopheles gambiae* s.s. population dynamics to better understand mosquito response to changes in biotic and abiotic variables. This model is then calibrated against longitudinal vector abundance data from Tanzania, before applying the methods of matrix population modelling to analyse model behaviour (specifically, adult *An. gambiae* s.s. response to environmental conditions), and, hence, assess the implications for the design and implementation of local vector control strategies.

## Results

A valuable modelling framework for assessing the effects of rainfall, cloudiness, wind speed, desiccation, temperature, relative humidity and density-dependence on vector abundance is developed, allowing ease of construction, analysis, and integration into malaria transmission models. Model calibration demonstrates excellent agreement with abundance data over a 40 month period, suggesting that recent malaria reductions in certain areas of Africa could be due to changing environmental conditions. Analysis of the model demonstrates the dependence on climatic conditions of *An. gambiae* s.s. persistence, resilience, stable stage distribution, and reproductive value of each of its lifecycle stages to fluctuations in local environmental conditions.

## Conclusions

Mathematical models provide a valuable means of understanding the role of environmental variables on malaria vectors and hence for better understanding future malaria transmission under different environmental conditions, as well as the potential impacts of climate change. Modelling and analysis here provides an important contribution to this goal, enabling direct assessment of the implications of climatic and environmental conditions on the design of optimal local vector control strategies, as well as evaluating the potential impact of climate change on the likely success of these interventions. The work also highlights research gaps and priorities that should be targeted if we are to more reliably understand future malaria risk in regions predicted to experience significant changes in climatic variables over the coming decades.

